# Analgesic effect of nitrous oxide during manual therapy after anterior cruciate ligament reconstruction: a study protocol for a randomized controlled trial

**DOI:** 10.1186/s13063-023-07732-z

**Published:** 2023-11-27

**Authors:** Ziyang Wang, Fei Wang, Yuxiang Li, Yihui Xing, Xiaochen Jiang, Cui Li, Zhiguo Ding, Lu Tang

**Affiliations:** 1 Department of Stomatology, the 960th Hospital of People’s Liberation Army of China (PLA), Jinan, Shandong China; 2 School of Nursing, Weifang Medical University, Weifang, Shandong China; 3 Department of Anesthesiology, the 960th Hospital of People’s Liberation Army of China (PLA), Jinan, Shandong China; 4 School of Nursing, Ningxia Medical University, Ningxia, Yinchuan, China; 5 Department of Rehabilitation Medicine, the 960th Hospital of People’s Liberation Army of China (PLA), Jinan, Shandong China; 6 Department of Hepatopancreatobiliary Surgery, Qingdao Municipal Hospital, Qingdao, Shandong China

**Keywords:** Nitrous oxide, Analgesia, Rehabilitation, Manual therapy

## Abstract

**Background:**

Many patients during manual therapy after anterior ligament reconstruction will experience severe pain, which has a negative impact on their rehabilitation. However, there is rarely an analgesic method for these patients during rehabilitation. Nitrous oxide with rapid analgesic and sedative effects is often used to relieve pain in minor procedures. The purpose of this study is to determine whether or not nitrous oxide analgesia decreases pain compared to oxygen during manual therapy after anterior ligament reconstruction.

**Methods/design:**

This single-center, randomized, double-blind and controlled trial will recruit 120 patients. Patients ≥ 18 years old undergoing manual therapy after anterior ligament reconstruction (1 month post-operative) with acute pain (VAS ≥ 4) are included. The main exclusion criteria included the following: pulmonary embolism, intestinal obstruction, pneumothorax. Patients will be randomly allocated to the intervention group (A) and the control group (B) in a ratio of 1:1. Doctors, therapists, patients, and data collectors are all blind to the study. The manual therapy will be performed by therapists. Nurses who implemented the intervention handed the doctors envelopes containing the patients’ codes and allocation of A or B. Group A will receive a pre-prepared nitrous oxide/oxygen mixture plus conventional treatment (no analgesic) given as 30-min treatment sessions, once daily, and group B will receive oxygen plus conventional treatment (no analgesic) under the same conditions. Assessments will be taken 2 min before the intervention (T0), 5 min after the beginning of the intervention (T1), and 5 min after the intervention finished (T2). The primary outcome is pain score. Secondary outcomes include vital signs, side effects, joint range of motion, adjuvant analgesia need, therapist and patient satisfaction, and whether willing to receive the same gas again.

**Expected outcomes:**

We expect nitrous oxide inhalation to have a beneficial effect on the pain of patients who receive manual therapy after anterior ligament reconstruction.

**Discussion:**

If this treatment appears beneficial, it could improve patients’ satisfaction and quality of life potentially and even be implemented widely in hospital and rehabilitation settings.

**Trial registration:**

ClinicalTrials.gov identifier, ChiCTR2200061175 (Version 2.0 June 15, 2022), https://www.chictr.org.cn.

**Supplementary Information:**

The online version contains supplementary material available at 10.1186/s13063-023-07732-z.

## Introduction

Anterior cruciate ligament (ACL) reconstruction is one of the most common operations in orthopedic [1]. ACL reconstruction after a ligament tear has been considered the key to treatment, especially for those wishing to resume normal life [2]. However, considerable postoperative complications such as pain, swelling, and a sudden impairment of motor function, muscle contracture, and joint stiffness may occur after ACL reconstruction surgery [1, 3]. Rapid return to normal function after surgery is a desirable result for patients and health providers. Rehabilitation is imperative to implement for alleviating pain and joint range of motion (ROM) gain [4]. Manual therapy (MT) after ligament reconstruction includes passive joint movement, joint stretching, free hand strength training, and other techniques, which are widely used in the postoperative recovery of various musculoskeletal diseases [5]. The effect of the technique can be attributed to the mechanisms of improving soft tissue ductility, stimulating peripheral mechanical receptors, and triggering neurophysiological responses [6, 7]. Rehabilitation after ACL reconstruction is painful as it generally begins early post-surgery. The experience of anxiety and fear also increases the perception of pain and even hinders the patients’ compliance with future treatment, which ultimately impacts surgical results [8]. Pain management during rehabilitation may reduce the incidence of complications, improve quality of life, shorten hospital stays, and reduce health care costs [9].

Oral or intravenous analgesics are usually used for immediate to short-term postoperative pain, but there is no systematic rehabilitation period analgesia program through literature review. Commonly used drugs include systemic opioids, non-steroidal anti-inflammatory drugs, and gabapentinoids [10, 11]. Although they can alleviate pain, it has many side effects concurrently, such as nausea and vomiting, lethargy and over-sedation, respiratory depression, and gastrointestinal tract damage [12]. These side effects may lead to delayed recovery after anesthesia or early postoperative recovery, increasing the length of stay and reducing patient satisfaction. Nitrous oxide (N_2_O), as an analgesic and sedative, which has the advantages of simple handling, fast onset, good effect, and few adverse reactions, is commonly used in various painful procedures [13]. Different studies have reported that the use of N_2_O can bring about notable pain relief in different areas of medicine such as minor dental surgery, emergency trauma, labor analgesia, plastic operation, colonoscopy, cancer pain, bone marrow biopsy, and invasive operation in pediatrics [14–21]. It usually produces effective and transient analgesia without changing consciousness and cognition, so patients can soberly cooperate with therapists during treatment. The device is portable and cheap, and patients can control it easily. It was observed that all side effects (dizziness, euphoria, nausea, vomiting, etc.) were temporary and disappeared within 5 min after stopping inhalation. No serious complications were found [22, 23].

There is an absence of a trial assessing the efficacy of N_2_O with MT after ACL reconstruction surgery. It is required that reasonable analgesia for patients who underwent MT. Thus, the purpose of this study is to evaluate the analgesic effect of inhaling N_2_O during rehabilitation on patients receiving MT through a randomized double-blind controlled trial. We hypothesized that its analgesic properties would decrease pain compared to placebo during MT with few side effects.

## Method

### Study design

**Fig. 1:**
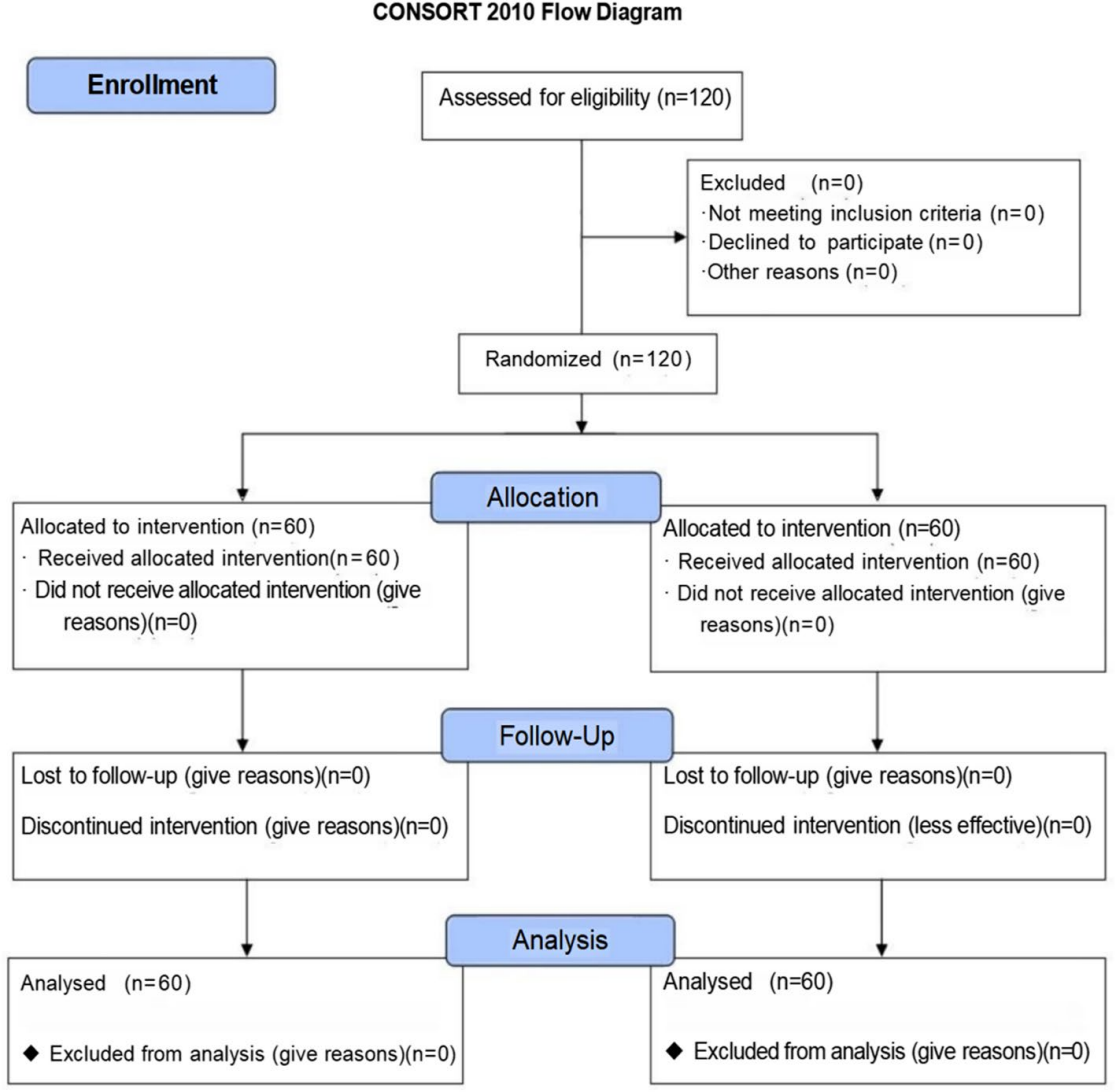
The CONSORT flow chart

The proposed clinical trial will be a single-center, randomized, double-blind, and controlled intervention study for the analgesic effect and safety of N_2_O during MT. It is registered in the Chinese Clinical Trial Registry (ChiCTR2200061175) and is in full adherence to the principles of the Declaration of Helsinki and Good Clinical Practice (GCP) guidelines. This trial will be conducted at the rehabilitation department of the 960th Hospital of PLA in China, and 120 patients will be randomly enrolled in two regimen arms with a ratio of 1:1; the recruitment will begin in July 2022. Although this study is a single-center, further multi-center clinical trials will be conducted in the future to provide more reliable evidence for the research results. The data collection phases of the CONSORT flow chart and the schedule of enrolment, and interventions are shown in Fig. 1 and Table 1, respectively.

**Table 1:** Schedule of inclusion, interventions and assessments

**Time points**	**Study period**					
	**Enrolment**		**Post-allocation**			**Close-out**
	Patients with acute pain caused by manual treatment after anterior ligament reconstruction		T0	T1	T2	Data collection measurements
**Patients**						
Eligibility screen	√					
Informed consent	√					
Allocation	√		√			
**Interventions**						
Intervention group				√		
Control group				√		
**Assessments**						
Pain score			√	√	√	Visual analogue scale
BP			√	√	√	Non-invasive electronic manometer
HR			√	√	√	Digital monitoring
SPO_2_			√	√	√	Digital monitoring
Side effects				√	√	Yes/no
ROM					√	Goniometer
Satisfaction					√	Satisfied/dissatisfied
Adjuvant analgesia					√	Yes/no
Acceptance					√	Yes/no

*Abbreviations*: *BP* blood pressure(mm/Hg), *HR* heart rate(bpm), *SPO*_*2*_ oxygen saturation(%), *ROM* range of motion(°)

### Ethics and dissemination

This protocol has been approved by the Ethics Committee (2021–59). We will protect the rights and safety of participants by full compliance with the Declaration of Helsinki and GCP guidelines. We will obtain written informed consent from the participants. Appropriate measures will be taken to ensure the confidentiality of data gathered in this study. The results of this study will be disseminated to the public through relevant academic and professional journals and conferences.

Any amendment to the protocol which may influence the conduct of the trial, the potential benefit or safety of the patient, including changes in study objectives, study design, patient population, sample sizes, or study procedures will require a formal modification to the protocol. Such modifications will be agreed upon by all members of the trial, and approved by the Ethics Committee before implementation and notified to the trial registries, journals, and regulators.

### Participants

All patients who received MT after ACL reconstruction facing acute pain will be invited to participate in this study. To decrease confounding factors that may interfere with pain scores during the rehabilitation, we will recruit patients with relatively consistent surgical site as much as possible (unilateral joint). The inclusion criteria are as follows: willing to participate in the trial and sign informed consent; man or woman; age ≥ 18 years old; patients underwent primary unilateral ACL reconstruction (1 month post-operative, with or without meniscal injury or collateral injury); experience worst pain (during MT) was reported ≥ 4 (on a visual analogue scale (VAS) of 0–10 cm); without postoperative complication. Exclusion criteria include the following: patients with pain unrelated to MT; patients who have undergone other surgical operations on the knee except for ACL; mental disorder or altered mental status have difficulty in reporting pain; patients with contraindications to N_2_O (abdominal distension or suspected bowel obstruction, air embolism, pneumothorax, decompression sickness, epilepsy, pulmonary cancer, chronic obstructive pulmonary disease and acute respiratory infection, pregnancy, severe inhalation injury, pharmaceutical or pathological pulmonary fibrosis, maxillofacial injuries, disease involving ear, nose, larynx, such as sinuses, otitis media); drug dependence or abuse. The main inclusion and exclusion criteria are presented in Table 2.

**Table 2:** Main inclusion and exclusion criteria

**Inclusion criteria**	**Exclusion criteria**
Patients with acute pain caused by manual treatment after anterior ligament reconstruction	Patients with mental disorder or altered mental status have difficulty in reporting pain
Age ≥ 18 years old	Patients who have undergone other surgical operations on the knee except for ACL
Willing to participate in the trial and sign informed consent	Drug dependence or abuse
Background worst pain (during Manual therapy) reported being 4 or higher (on a visual analogue scale (VAS) of 0 to 10 cm)	Contraindications to N_2_O (suspected bowel obstruction, air embolism, pneumothorax, epilepsy, chronic obstructive pulmonary disease, and acute respiratory infection, pregnancy, pharmaceutical or pathological pulmonary fibrosis, maxillofacial injuries, disease involving ear, nose, larynx)
Without postoperative complication	

### Recruitment strategies

Potential participants for the study will be sourced from the 960th Hospital of PLA, which is a general hospital in Shandong. Recruitment sources will be focused on patients who undergoing MT after ACL reconstruction. A researcher of the team will contact the case managers, participants, and other relevant staff for information about the study. Researchers will introduce the details to patients so that they can fully understand the potential benefits and risks of participating in this study. If that patient is interested in participating, they will voluntarily contact the researcher and complete a “consent to contact form.” Special researchers assess that the participants who meet the inclusion criteria will sign the “informed consent form.” The two gases will be provided to the participants free of charge. Participants will be provided with professional medical services and disease assessment by medical workers throughout the study period to encourage patients to complete the entire study. Appropriate compensation will be given to the participants for the inconvenience of time or any harm by the trial.

### Randomization, allocation, concealment and blinding

To minimize selective bias, a member of our research team is not involved in any other section of the study. Eligible patients will be randomly divided into the intervention and control groups with a ratio of 1:1 to receive: 30-min inhalation of pre-prepared nitrous oxide/oxygen mixture plus conventional treatment (no analgesic) or inhalation of placebo consisting of oxygen plus conventional treatment (no analgesic) for 30 min. Before initiating recruitment, the randomization sequence is generated and validated by a statistician using Microsoft Excel and then kept by an administrator who does not participate in this study. The list will be sealed in an envelope and stored in a designated room to ensure concealment. After participants meet all the inclusion criteria and complete the baseline assessment, the project manager will apply for random numbers and the patients will receive the corresponding treatment. Except for the project manager, who is responsible for gas distribution, other researchers, operators, and patients do not know the allocation, to ensure the implementation of blinding. In addition, considering that two gases will significantly affect the outcome of this trial, all cylinders with the same appearance containing N_2_O and O_2_ will be covered with labels A and B until completing all the procedures of this trial.

To maintain the overall quality and legitimacy of the study, code breaks should occur only in exceptional circumstances such as serious side effects when knowledge of the actual treatment is essential for further management of the patient. If unblinding is deemed to be necessary, the researcher should use the emergency system for unblinding.

### Intervention

After inclusion in the study, participants will be randomized into intervention and control groups. All procedures will be carried out in the rehabilitation treatment hall. MT includes passive joint movement and joint stretching for a total of 30 min. The procedure is performed by qualified rehabilitation therapists who have worked for more than 2 years in the hospital. And the two gases implemented are conducted by trained and qualified nurses. Before and after the MT, wax therapy, and cold compress are applied as routine treatments. The patients in the intervention group began inhaling pre-mixed 65% N_2_O through a specially designed mask with a check valve, 2 min before MT, and continue the whole procedure until it ends, while the control group inhaled 100% oxygen under the same conditions. The gas will be administered by the patients themselves in order to observe their state of consciousness. Blood pressure will be measured before, during, and after each MT, and the oxygen saturation and heart rate were continuously monitored with a pulse oximeter throughout the process. Image and audio recordings will be taken individually for communication coding and data analysis. Adjuvant analgesics will also be recorded.

### Outcome measures

#### Baseline data

The demographic data of all participants (gender, age, height, weight, country, nationality and occupation, etc.) and clinical characteristics (including type and location of damage, current pain level) will be recorded with an internally designed form at baseline (T0, 2 min before the intervention). Vital signs such as blood pressure (BP), heart rate (HR), and oxygen saturation (SPO_2_) will also be collected at baseline.

#### Primary outcome

The primary outcome measure will be the degree of pain assessed on the VAS (a patient self-assessment scale) at T0, T1 (5 min after the beginning of the intervention), and T2 (5 min after the intervention finished), respectively. The time point selection was based on the pharmacokinetics of N_2_O. The VAS is a horizontal line without numbers or verbal descriptors at intermediate points, ranging from 0 to 10 cm (0: no pain; 10: worst imaginable pain). The VAS is chosen due to its simplicity, adaptability to a broad range of populations, and more important, it has good visual properties [24].

#### Secondary outcome

Based on previous studies [19, 25, 26], secondary outcomes cover vital signs at T0, T1, and T2 to observe the impact of pain and intervention measures on the patient’s physiological index. The side effects, patient and therapist satisfaction (0: satisfied; 1: dissatisfied), the time for ROM ≥ 90° (according to our previous qualitative study, one of the substantial indicators of discharge from hospital for patients is range of motion ≥ 90°), and whether to add adjuvant analgesia will be obtained at T2 separately. Whether patients are willing to use the same gas again should also be recorded as an indicator and recorded.

### Sample size estimates

A prospective sample size estimate was conducted by a senior statistician using the GPower 3.1 procedure during the protocol-writing stage. The level of pain relief is regarded as the primary endpoint measure of this trial [27]. To achieve a statistical power of 80% (*β* = 0.20) and two-tailed testing (at a type-1 error rate of 5%) is sufficient. Based on the pre-trial with 20 patients (Corr among rep measures = 0.602), a sample size of 12 was targeted as being sufficient to achieve the required effective sample size, but to meet the Chinese Food and Drug Administration standard for the safety and feasibility of nursing staff implementing this inhalation analgesic, the targeted sample size of 120 was recommended.

### Data management and analysis

Data are treated confidentially. To improve the quality of data collection, all researchers will receive professional training before the study. All study-related information (e.g., data collection forms/digital files) will be stored securely in the medical office. Personally identifiable information of participants, such as names, and gender, will be replaced by codes throughout the study and will always remain confidential, with only the project manager or relevant doctors aware of the patient’s personal information.

And any public reports on the results of this study will not reveal any identifiable information about the patient. Composition of data monitoring committee (DMC) and auditing system will be established shortly after the project launch to monitor the collection and management of data and patient safety throughout the study. Members will include four professional therapists and nurses, two pain management specialists, and a senior academic statistician who worked as the board’s chair.

A double data check has also been performed for quality control. According to the data collection methods and criteria developed by the project leader and professionals, all data will be recorded carefully and comprehensively to ensure the authenticity of the data. Missing data on withdrawn participants will be imputed using the multiple imputation method.

All statistical analyses will be performed by SPSS version 25 (IBM Inc, Armonk, New York), with an alpha set at 0.05. Statisticians will calculate the mean (standard deviation), median (interquartile range), minimum, maximum, and proportion (95% confidence interval) for quantitative data and describe numbers and percentages for qualitative data. The mean will be compared by Student’s *t*-test (normal distribution parameter) or nonparametric two-sample Mann–Whitney test (nonnormal distribution parameter). The proportions will be compared using the chi-square test or Fisher exact test. The comparisons between two groups will be performed as follows: *t*-tests or Wilcoxon rank sum (normal or nonnormal ability distributions) for quantitative data; chi-square test or Fisher’s exact test for qualitative data. The value of *P* < 0.05 will be considered statistically significant.

### Safety and AE monitoring

Side effects include dizziness, headache, nausea, vomiting, lethargy, euphoria, oxygen destruction (pulse oxidative saturation ≤ 94%), arterial hypotension, bradycardia, or any other uncomfortable reactions that will be assessed and recorded during and after the procedure. In case of any of the above conditions, the inhalation of gas will be stopped immediately and oxygen will be given to the patient, then the discomfort will rapidly disappear within 5 min [28, 29].

### Quality control

The research will be coordinated and supervised by pain management specialists, rehabilitation therapists, nurses, and senior statisticians. To ensure the quality of the study, at the beginning of this study, the project manager and team members formulated a detailed project protocol and emergency plan. During the trial, researchers will receive unified and targeted training to ensure the smooth implementation of the study. The study funders will use the audits to regularly check the source of the data and its accuracy, integrity, reliability, and security.

### Role of the funding source

The funder had no role in the study design, data collection, analysis, interpretation, or report writing. The corresponding author had full access to all the data in the study and had final responsibility for the decision to submit for publication.

## Discussion

From an orthopedic perspective, postoperative rehabilitation plays an important role in maximizing the patients’ functionality and helping the management of these potential post-operative problems [30]. However, MT is conducted when the patient is conscious. Pain may be a major obstacle to MT sessions in this population [31, 32]. Proper pain management and timely ROM acquisition after surgery can not only improve patients’ satisfaction and quality of life but also reduce the burden on nursing staff and the economic burden of the patient group on health and social care services.

Our unpublished preliminary qualitative interview indicated that patients were asked to endure severe pain but no analgesics were supplied. The reasons for poor pain management may include medical workers’ and patients’ resistance to opioids, inappropriate cognitive understanding of pain, and lack of systematic pain management education [33–35].

The appropriate analgesics are integral for pain control postoperative of ligament reconstruction. Compared with traditional opioid analgesics, N_2_O is a self-administered inhaled gas reserved in a pre-prepared cylinder and has the characteristics of analgesia, sedation, and anti-anxiety. It has been widely used for nearly a century in other countries, especially in Britain, Sweden, the Netherlands, and Australia. The use rate of nitrous oxide is as high as 80% [36], which is mainly used in clinical disciplines such as dentistry, emergency, obstetrics and gynecology, pediatrics, and postoperative analgesia. It is reported that the mixture of 20% N_2_O and 80% O_2_ has an equivalent analgesic effect as well as a good sedative effect with 15 mg morphine [37]. Notably, it does not combine with hemoglobin and is mainly discharged through the lungs with a strong analgesic effect but a weak anesthetic effect [31, 38].

Therefore, we designed this study to explore the efficiency of N_2_O for patients receiving MT after ACL reconstruction. If feasible, it will be widely applied in rehabilitation training after ACL reconstruction and improve the quality of life and satisfaction of patients.

## Limitation

Some limitations must be acknowledged in this study. Firstly, the pain score was the subjective outcome and was not consistently assessed by individual patients; therefore, it is susceptible to the subjective influence of patients. Secondly, data extraction is only during and after the procedure, without long-term effect records.

## Conclusion

To our best knowledge, this study is the first randomized controlled trial to evaluate the effectiveness of the N_2_O to treat acute pain in patients undergoing MT after ACL reconstruction. If this treatment appears beneficial, this study can help to generate preliminary guidelines on pain management in patients undergoing MT. We will disseminate the results of this study to international journals and conferences.

## Trial status

This trial has been registered in the clinical trial registry: ChiCTR2200061175 (Version 2.0 June 15, 2022). Participant recruitment began on June 27, 2022, and is expected to be completed within 1 year. At the time of writing the manuscript, a total of 98 participants had been included in the study. The participating clinical centers are positively recruiting and collecting data.

### Supplementary Information

**Additional file 1: [S1. SPIRITR2].** Standard Protocol Items: Recommendations for Interventional Trials (SPIRIT) Checklist.

## Data Availability

After the completion of the study, the final trial data and statistical code will be obtained from the project leader for reasonable reasons. No project member can get the data privately.

## Abbreviations

ACL: Anterior cruciate ligament
MT: Manual therapy
N_2_O: Nitrous oxide
O_2_: Oxygen
HR: Heart rate
BP: Blood pressure
SPO_2_: Oxygen saturation
VAS: Visual analogue scale
ROM: Joint range of motion
